# An Increase in Antibiotic Prescribing for Respiratory Tract Infections Through Telehealth Consultations: Retrospective Study in Australian General Practice

**DOI:** 10.2196/40876

**Published:** 2022-10-18

**Authors:** Chisato Imai, Janaki Amin, Mirela Prgomet, Christopher Pearce, Andrew Georgiou

**Affiliations:** 1 Australian Institute of Health Innovation Macquarie University Sydney Australia; 2 Department of Health Sciences Macquarie University Sydney Australia; 3 Aurora Primary Care Research Institute Blackburn Australia

**Keywords:** general practice, anti-infective agents, antibiotics, medication, prescriptions, respiratory tract infections, infection, telehealth, telemedicine, Australia

## Introduction

General practitioners (GPs) are the first point of contact for most people in Australia seeking medical attention. They play an important role in the prevention, early detection, and management of both acute and chronic diseases. In Australian general practice, antibiotics are commonly prescribed for respiratory tract infections (RTIs) despite evidence of limited efficacy [1,2]. A previous study identified that the antibiotic prescribing rates for RTIs by GPs are up to 9 times higher than recommended by practice guidelines [3]. Such findings have raised public health concerns about overprescribing antibiotics, emphasizing the need for continued monitoring of antibiotic prescribing activities in Australian general practice.

In mid-March 2020, the Australian government implemented the expansion of telehealth services covered by Medicare (Australia’s universal health insurance) in response to the COVID-19 pandemic. Subsequently, many GPs shifted care delivery from face-to-face consultations to telehealth (telephone or video-conference consultations). A study conducted in the early stage of the pandemic showed lower rates of medication prescriptions via telehealth compared to face-to-face consultations in Australian general practice [4]; however, antibiotic prescribing for RTIs by consultation modality is yet to be explored. Therefore, we examined antibiotic prescribing for RTIs via telehealth in comparison with face-to-face consultations from April 2020 to November 2021.

## Methods

### Data and Analysis

This retrospective study used data from the Population Level Analysis and Reporting (POLAR) platform [4]. POLAR comprises deidentified electronic health records collated from approximately 1000 general practices across 2 states in Australia (ie, Victoria and New South Wales). To obtain our variables of interest, diagnosis data were used to identify consultations for respiratory infections based on SNOMED-CT (Systematized Nomenclature of Medicine – Clinical Terms) codes (respiratory infection, [viral/bacterial/recurrent/acute] upper respiratory tract infection, and [viral/bacterial/recurrent/acute] lower respiratory tract infection); antibiotic prescriptions were identified via the Anatomical Therapeutic Chemical code J01 (antibacterials for systemic use) in prescription data; and consultation modality was defined based on service item numbers from Medicare billing data [5].

For the analysis, we examined the mean weekly percentage of consultations with antibiotic prescribing between April 1, 2020, and November 30, 2021. We also determined the patient-level probability of an antibiotic being prescribed during consultations in the periods June to November 2020 and June to November 2021, using generalized estimating equations with the Huber-White standard error, adjusted for patient factors (age, gender, remoteness, state of residence, active status as defined by the Royal Australian College of General Practitioners [RACGP], and recent history of consultation) and a sampling effect within the general practices. Active patients (ie, regular visitors to a practice) were defined based on the definition from the RACGP as individuals who had attended the practices 3 or more times in the past 2 years at the time of visit [6].

### Ethics Approval

Ethics approval was obtained from the Macquarie University Human Research Ethics Committee (#52020675617176).

## Results

Between April 2020 and November 2021, a total of 105,719 individuals had an RTI diagnosis and attended 141,444 consultations: 92,318 (65.3%) face-to-face visits and 49,126 (34.7%) telehealth consultations (comprising 48,159 via telephone and 967 via video conference).

**Figure 1:**
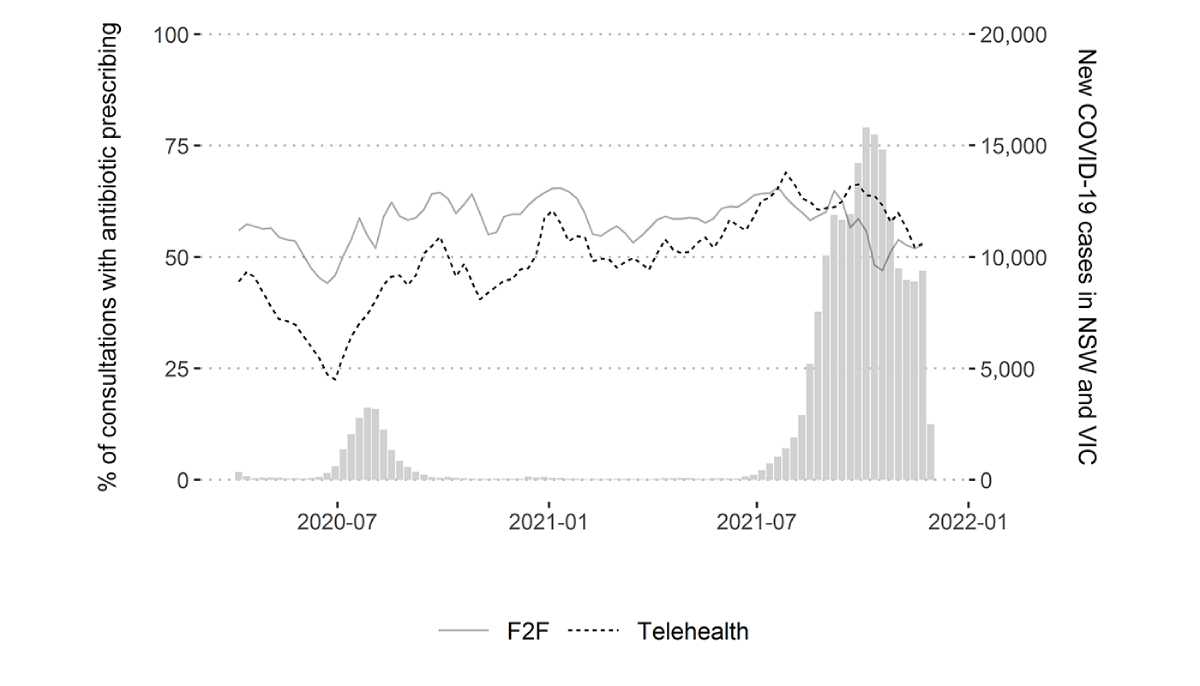
Weekly mean percentage of respiratory tract infection consultations with an antibiotic prescription for F2F and telehealth consultations (April 2020 to November 2021). F2F: face-to-face; NSW: New South Wales; VIC: Victoria.

In 2020, the weekly mean antibiotic prescribing rates for RTIs were 56.7% (95% CI 54.2%-59.3%) for face-to-face consultations and 40.8% (95% CI 37.2%-44.4%) for telehealth (Figure 1). In 2021, the weekly mean prescribing rates were 58.6% (95% CI 55.8%-61.4%) for face to face and 61.0% (95% CI 59.1%-63.0%) for telehealth. We also evaluated the number of prescriptions during the study period and observed the same longitudinal trend.

At the patient level, the probability of receiving an antibiotic prescription through a telehealth consultation also increased from 59.3% (95% CI 57.6%-61.0%) in 2020 to 65.7% (95% CI 64.4%-67.0%) in 2021 (Table 1). The probability via face-to-face consultations was consistent across 2020 and 2021: 65.2% (95% CI 63.4%-66.9%) and 66.9% (95% CI 65.5%-68.2%), respectively.

**Table 1:** Patient probability of receiving antibiotic prescriptions in a consultation for respiratory tract infection.

Variables		Estimated probability (%) of receiving antibiotic prescription (95% CI)	
		2020	2021
**Age group (years)**			
	<10	54.2 (52.0-56.6)	54.7 (53.0-56.4)
	10-24	50.5 (47.6-53.6)	59.1 (56.3-62.0)
	25-44	61.6 (58.9-64.5)	67.7 (65.9-69.6)
	45-64	74.6 (71.2-78.1)	73.2 (71.0-75.5)
	≥65	93.4 (87.7-99.4)	83.3 (80.3-86.5)
**Sex**			
	Female	65.2 (63.4-66.9)	66.9 (65.5-68.2)
	Male	58.0 (56.2-59.8)	65.6 (64.1-67.1)
**Active status**			
	Nonactive	48.9 (47.2-50.7)	51.9 (50.3-53.6)
	Active	65.2 (63.4-66.9)	66.9 (65.5-68.2)
**Remoteness**			
	Major cities	65.2 (63.4-66.9)	66.9 (65.5-68.2)
	Regional or remote areas	75.8 (72.1-79.7)	77.3 (74.9-79.7)
**Previous encounter (≤2 weeks)**			
	No	65.2 (63.4-66.9)	66.9 (65.5-68.2)
	Yes	63.6 (61.9-65.3)	66.5 (65.2-67.9)
**State**			
	New South Wales	65.2 (63.4-66.9)	67.7 (66.4-69.1)
	Victoria	54.6 (52.9-56.4)	66.9 (65.5-68.2)
**Consultation**			
	Face to face	65.2 (63.4-66.9)	66.9 (65.5-68.2)
	Telehealth	59.3 (57.6-61.0)	65.7 (64.4-67.0)

## Discussion

Antibiotic prescribing via telehealth increased over time, with rates initially much lower than face-to-face consultations; however, the prescribing rates between the two consultation modalities became equivalent toward the end of 2021.

While telehealth offers some advantages such as less travel time and prevention of infectious disease transmissions, the caveats include limited physical examination capabilities. Such limitations may potentially impact the adequacy of medication prescribing, particularly in populations like children who have difficulty verbalizing symptoms.

Considering that high antibiotic prescribing rates for RTIs by Australian GPs is a long-standing public health concern, the findings from this study highlight the need for monitoring the impacts of telehealth on medication prescribing in general practice. Further, studies on telehealth decision-making processes for antibiotic prescribing that evaluate prescribing adequacy appear critical.

## Abbreviations

GP: general practitioner
POLAR: Population Level Analysis and Reporting
RTI: respiratory tract infection
RACGP: Royal Australian College of General Practitioners
SNOMED-CT: Systematized Nomenclature of Medicine – Clinical Terms
